# Rapid real‐world data analysis of patients with cancer, with and without COVID‐19, across distinct health systems

**DOI:** 10.1002/cnr2.1388

**Published:** 2021-05-20

**Authors:** Clara Hwang, Monika A. Izano, Michael A. Thompson, Shirish M. Gadgeel, James L. Weese, Tom Mikkelsen, Andrew Schrag, Mahder Teka, Sheetal Walters, Frank M. Wolf, Jonathan Hirsch, Donna R. Rivera, Paul G. Kluetz, Harpreet Singh, Thomas D. Brown

**Affiliations:** ^1^ Henry Ford Cancer Institute Henry Ford Health System Detroit Michigan USA; ^2^ Syapse San Francisco California USA; ^3^ Aurora Cancer Care Advocate Aurora Health Milwaukee Wisconsin USA; ^4^ Oncology Center of Excellence United States Food and Drug Administration Silver Spring Maryland USA

**Keywords:** cancer risk factors, epidemiology, medical oncology, viral infection

## Abstract

**Background:**

The understanding of the impact of COVID‐19 in patients with cancer is evolving, with need for rapid analysis.

**Aims:**

This study aims to compare the clinical and demographic characteristics of patients with cancer (with and without COVID‐19) and characterize the clinical outcomes of patients with COVID‐19 and cancer.

**Methods and Results:**

Real‐world data (RWD) from two health systems were used to identify 146 702 adults diagnosed with cancer between 2015 and 2020; 1267 COVID‐19 cases were identified between February 1 and July 30, 2020. Demographic, clinical, and socioeconomic characteristics were extracted. Incidence of all‐cause mortality, hospitalizations, and invasive respiratory support was assessed between February 1 and August 14, 2020. Among patients with cancer, patients with COVID‐19 were more likely to be Non‐Hispanic black (NHB), have active cancer, have comorbidities, and/or live in zip codes with median household income <$30 000. Patients with COVID‐19 living in lower‐income areas and NHB patients were at greatest risk for hospitalization from pneumonia, fluid and electrolyte disorders, cough, respiratory failure, and acute renal failure and were more likely to receive hydroxychloroquine. All‐cause mortality, hospital admission, and invasive respiratory support were more frequent among patients with cancer and COVID‐19. Male sex, increasing age, living in zip codes with median household income <$30 000, history of pulmonary circulation disorders, and recent treatment with immune checkpoint inhibitors or chemotherapy were associated with greater odds of all‐cause mortality in multivariable logistic regression models.

**Conclusion:**

RWD can be rapidly leveraged to understand urgent healthcare challenges. Patients with cancer are more vulnerable to COVID‐19 effects, especially in the setting of active cancer and comorbidities, with additional risk observed in NHB patients and those living in zip codes with median household income <$30 000.

## 1 INTRODUCTION

Since the first cases of a pneumonia of unknown etiology in Wuhan, China, were reported in late 2019, more than 9 million confirmed cases of the novel coronavirus identified as severe acute respiratory syndrome coronavirus 2 (SARS‐CoV‐2) and over 231 000 related deaths have occurred in the United States.^1^ Questions remain about the clinical epidemiology of coronavirus disease (COVID‐19), including the characteristics of infected populations and the factors that influence susceptibility and disease severity or mortality risk, including the need for intensive care unit (ICU) care or mechanical ventilation.

In the United States, various reports suggest that COVID‐19 incidence and related outcomes vary by race, ethnicity, and socioeconomic status.^2^, ^3^, ^4^, ^5^, ^6^ Higher incidence of COVID‐19 infection has been reported among black and Latino Americans^2^, ^3^, ^6^ and in counties with either more diverse populations or a higher proportion of adults with less education than a high school diploma.^6^ Higher mortality has also been reported in counties in which black Americans comprise a larger proportion of the population or counties with larger percentages of residents living below the poverty level, on Medicaid, or living with a disability.^6^, ^7^ The constellation of factors associated with increased rates and/or severity of COVID‐19 infection in under‐represented minority populations reinforce the role that social determinants of health play in health outcomes.

Patients with a history of cancer and those undergoing active treatment for malignancies who contract COVID‐19 are also susceptible to poor outcomes. Selected studies conducted in New York and Northern California have estimated that cancer was present in a minority (5%‐6%) of hospitalized COVID‐19 patients^8^, ^9^, ^10^; yet research indicates that cancer in patients diagnosed with COVID‐19 is associated with increased risk of severe events (ICU admission, mechanical ventilation, death), with greater risk among patients with metastatic disease, recent treatment (past month) with chemotherapy, immune checkpoint inhibitor therapy (90 days), or surgery.^10^, ^11^, ^12^, ^13^, ^14^, ^15^, ^16^, ^17^ Additionally, quicker progression to severe events was found among patients with cancer than among those without cancer.^14^, ^18^, ^19^

In the United States, data characterizing COVID‐19 in patients with cancer are still quite limited and generally obtained within single health systems or from voluntary surveillance registries or surveys.^10^, ^12^, ^13^, ^20^, ^21^, ^22^, ^23^, ^24^, ^25^ The impact of race, health status, and socioeconomic factors with COVID‐19‐related incidence or outcomes in patients with cancer is not well described. To expand the available evidence, the current study utilizes the ability for rapid COVID‐19 case identification through access to integrated, detailed, longitudinal clinical data from two large Midwestern health systems to examine the differential risk for infection and severe outcomes among adults with a history of cancer and those undergoing active cancer treatment.

## 2 METHODS

### 2.1 Study population

This retrospective observational cohort study included patients who received cancer treatment or ongoing surveillance between May 15, 2015 and February 1, 2020 at two community health systems in the Midwestern United States. These health systems include over 700 sites of care (including 32 hospitals) to serve over 4 million patients across Illinois, Michigan, and Wisconsin. Integrated data that included electronic medical records (EMR) were used to identify the study population of patients with an *International Classification of Diseases, Tenth Revision, Clinical Modification* (ICD‐10‐CM) code of C00‐C99 for malignant cancer in EMR encounter records or synoptic pathology reports. Patients with active cancer were defined as those with a first encounter with ICD‐10 code for malignant neoplasm, or receipt of an anticancer agent within 12 months prior to February 1, 2020; patients with history of cancer were defined as those with encounters with an active cancer code from May 15, 2015, to February 1, 2019, and no receipt of anticancer therapy within the prior 12 months.

To ensure proper ascertainment of baseline medical history, patients whose first recorded encounter was within the 12 months prior to February 1, 2020, were excluded from this analysis. Among our study population of patients with cancer, we identified those who had a COVID‐19 diagnosis code in the EMR encounter records (ICD‐10‐CM: U07.1, B97.21, B97.29, J12.81, B34.2) and/or a positive 2019‐nCoV RNA, SARS CoV2 RNA‐RT PCR, or SARS‐CoV‐2‐Qual RT PCR laboratory test result between February 1, 2020, and July 30, 2020.

### 2.2 Outcome measures

Patients were followed from February 1, 2020, (the index date) to August 14, 2020. The primary outcome of this study was all‐cause mortality. Dates of death were obtained from the integrated health system data directly if available or from linkage to hospital tumor registries, digitized obituaries, the Social Security Death Index (SDI), and chart abstraction conducted by Certified Tumor Registrars (CTRs). Hospital tumor registries were accessed through MetriQ and CNExT. Secondary outcomes included hospitalization and utilization of invasive mechanical ventilation in the inpatient setting, the latter reported among the subset of 918 patients for which these data were available.

### 2.3 Covariates

Patient demographic and clinical characteristics evaluated include: cancer status (active vs history of cancer); age at the index date (<50, 50‐64, ≥65); sex (male, female); race/ethnicity: Non‐Hispanic white (NHW), Non‐Hispanic black (NHB), Hispanic/Latino, Asian/Native Hawaiian or Other Pacific Islander, and Other/Unknown; estimated median annual household income; comorbidity burden, hospitalization, or surgery (cancer‐related or otherwise) within the 12 months prior to the index date; select medication use in the 90 days prior to the index date; cancer type (hematological malignancy or solid tumor); receipt of antineoplastic treatment with immune checkpoint‐inhibitors, chemotherapy, or targeted therapies in the 30 days prior to the index date; participating health system; number of inpatient, outpatient or emergency department (ED) visits during follow‐up; common hospital admission diagnoses and discharge reasons; acute complications (described below), and COVID‐related treatments received in the inpatient setting for hospitalizations during the follow‐up period.

The estimated median household income in each residential zip code was derived from the 2010 US census (<$30 000, $30 000‐$60 000, $60 000‐$100 000, $100 000‐$185 000, >$185 000, unknown). Overall comorbidity burden was assessed with the Charlson‐comorbidity index, Quan version (CCI: 0, 1, ≥2).^26^ Hypertension, diabetes, chronic pulmonary disease, chronic kidney disease (CKD, grade 3 or 4), renal failure, liver disease, peripheral vascular disease, coagulopathy, pulmonary circulation disorders, obesity, HIV/AIDS, and rheumatoid arthritis/collagen vascular diseases were also extracted individually using ICD‐10 CM Codes.

For patients that were hospitalized, inpatient treatment with the following medications was assessed: vasopressors; azithromycin or other antibiotics; hydroxychloroquine, remdesivir, or other antiviral drugs; famotidine; and tocilizumab. We evaluated the following acute COVID‐19 complications: respiratory distress or failure; sepsis; renal failure; kidney injury; liver injury; arrhythmia; conduction disorders; cardiac arrest; cardiomyopathy, myocarditis, pericarditis; coagulopathy; chronic pulmonary disease; and cytokine release syndrome.

### 2.4 Statistical analysis

Demographic, clinical, and socioeconomic characteristics of patients with active cancer or history of cancer and COVID‐19 were compared to the characteristics of patients without recorded COVID‐19. We used multivariable logistic regression to estimate odds ratios (OR) and evaluate the association between clinical factors and all‐cause mortality. Models were adjusted for sex, age, race/ethnicity, median household income, CCI, recent surgery, hypertension, coagulopathy, pulmonary circulation disorders, obesity, cancer type, cancer status, and cancer treatment type. All analyses were performed in R programming language, version 3.3.2 (R Foundation for Statistical Computing, Vienna, Austria). A *P*‐value of <.05 was considered statistically significant.

## 3 RESULTS

The study population consisted of 147 969 patients with cancer (Table 1). Compared to patients without COVID‐19 (n = 146 702), a greater proportion of patients with COVID‐19 (n = 1267) were NHB, lived in a zip code with median annual household income of <$30 000, had a CCI of 2 or greater, had diabetes, or had active cancer. All‐cause mortality (14% vs 2%), incidence of hospital admission (64% vs 14%), and use of invasive respiratory support (11% vs 1%) were significantly greater among patients with cancer and COVID‐19 compared to patients with cancer without COVID‐19.

**TABLE 1:** Demographic, clinical, and socioeconomic characteristics of the study population of patients with cancer, with and without COVID‐19

	COVID‐19 (N = 1267)	No COVID‐19 (N = 146 702)
Demographic characteristics		
Age, Median (IQR)	66 (55, 75)	67 (57, 76)
Age categories, N (%)		
<60	182 (14%)	19 263 (13%)
60‐69	415 (33%)	42 963 (29%)
≥70	670 (53%)	84 476 (58%)
Sex, N (%)		
Male	539 (43%)	62 650 (43%)
Female	728 (57%)	84 049 (57%)
Missing	0 (0%)	3 (0%)
Race/ethnicity, N (%)		
Non‐Hispanic white	559 (44%)	99 591 (68%)
Non‐Hispanic black	425 (34%)	14 667 (10%)
Hispanic/Latino	54 (4%)	3690 (3%)
Asian/Native Hawaiian or Pacific Islander	19 (1%)	1489 (1%)
Other or unknown	210 (17%)	27 265 (19%)
Annual household income, median (IQR)		
0‐30 k	189 (15%)	6959 (5%)
30‐60 k	723 (57%)	80 005 (55%)
60‐100 k	315 (25%)	54 090 (37%)
100‐185 k	34 (3%)	4875 (3%)
185 k+	0 (0%)	1 (0%)
Missing	6 (0%)	772 (1%)
Clinical characteristics		
Comorbidities in the year prior to 2/1/2020, N (%)		
Charlson Comorbidity Index		
Median, IQR	1.0 (0, 2.0)	1.0 (0, 1.0)
0	426 (34%)	67 969 (46%)
1	379 (30%)	43 674 (30%)
2+	462 (36%)	35 059 (24%)
Hypertension	411 (32%)	39 901 (27%)
Diabetes	301 (24%)	19 354 (13%)
Chronic pulmonary disease	141 (11%)	11 680 (8%)
Grade 3/4 chronic kidney disease	125 (10%)	9118 (6%)
Renal failure	179 (14%)	11 771 (8%)
Liver disease	59 (5%)	3755 (3%)
Peripheral vascular disorders	77 (6%)	7125 (5%)
Coagulopathy	48 (4%)	3282 (2%)
Pulmonary circulation disorders	48 (4%)	2719 (2%)
Obesity	98 (8%)	7207 (5%)
HIV/AIDS	5 (0%)	209 (0%)
Rheumatoid arthritis/collagen vascular diseases	53 (4%)	3371 (2%)
Surgery in the year prior to 2/1/2020, N (%)	461 (36%)	41 176 (28%)
Cancer‐related characteristics		
Active cancers, N (%)	719 (57%)	69 924 (48%)
History of cancer, N (%)	548 (43%)	76 778 (52%)
Cancer type, N (%)		
Hematological	190 (15%)	18 426 (13%)
Breast	171 (13%)	23 576 (16%)
Genitourinary		
Prostate	153 (12%)	19 608 (13%)
Other	40 (3%)	6971 (5%)
Lung and respiratory		
Lung	80 (6%)	6514 (4%)
Other	0 (0%)	51 (0%)
Gastrointestinal		
Colorectal	33 (3%)	3394 (2%)
Other	74 (6%)	7213 (5%)
Abdominal	4 (0%)	708 (0%)
Gynecological	137 (11%)	13 914 (9%)
Bone and soft tissue	46 (4%)	4068 (3%)	
CNS and brain	10 (1%)	1207 (1%)	
Endocrine	53 (4%)	5491 (4%)	
Skin	18 (1%)	3619 (2%)	
Head and neck	19 (1%)	3144 (2%)	
Malignant, unknown site	19 (1%)	1286 (1%)	
Neoplasm, NOS	220 (17%)	27 460 (19%)	
Cancer treatment in the 30 days prior to 2/1/2020, N (%)			
Immune checkpoint inhibitors	12 (1%)	624 (0%)	
Chemotherapy	39 (3%)	2310 (2%)	
Targeted therapies	63 (5%)	3887 (3%)	
Clinical endpoints		
Mortality, N (%)	173 (14%)	3417 (2%)
Hospital admissions, N (%)	913 (72%)	54 057 (37%)
Invasive respiratory support, N (%)^a^	138 (15%)	790 (1%)

^a^ Among those for whom data were available.

### 3.1 Patients with cancer and COVID‐19

Among patients with cancer and COVID‐19, patients with active cancer were more likely than patients with a history of cancer to be male (48% vs 35%) or have a CCI of 1 or greater (79% vs 51%) (Table 2). Patients living in areas with median household income below $30 000 were more likely to be diagnosed with COVID‐19 by ICD codes alone (62% vs 49%). Among patients with COVID‐19, the incidence of death, hospital admission, and use of invasive respiratory support was greatest among patients with active cancer, or those residing in zip codes with median household income below $30 000; NHB patients were more likely to receive invasive respiratory support than the other racial/ethnic groups (Table 2). Females were less likely to have active cancer or comorbidities or to use antihypertensive medications. Females were also younger than males, and at lower risk of mortality, hospital admission, and of requiring invasive respiratory support (Table S1).

**TABLE 2:** Clinical characteristics of the patients with cancer and COVID‐19, by select characteristics

	Cancer Status		Median Household Income		Race/ethnicity		
	Active Cancers	History of Cancer	0‐30 k	>30 k	Non‐Hispanic White	Non‐Hispanic Black	Hispanic Latino
	N = 719	N = 548	N = 189	N = 1072	N = 559	N = 425	N = 54
COVID‐19 diagnosis, N (%)							
Positive test result only	479 (82%)	285 (86%)	129 (88%)	630 (83%)	344 (82%)	268 (84%)	33 (73%)
ICD code only	352 (60%)	202 (61%)	113 (77%)	441 (58%)	240 (57%)	230 (72%)	31 (69%)
Positive test result and ICD code	335 (57%)	173 (52%)	50 (34%)	453 (59%)	254 (61%)	142 (44%)	26 (58%)
Clinical characteristics							
Comorbidities in the previous year, N (%)							
Charlson Comorbidity Index							
Median, IQR	1.0 (1.0, 2.0)	1.0 (0, 2.0)	1.0 (0, 2.0)	1.0 (0, 2.0)	1.0 (0, 2.0)	1.0 (0, 2.0)	1.0 (0, 2.0)
0	155 (22%)	271 (49%)	66 (35%)	359 (33%)	174 (31%)	144 (34%)	15 (28%)
1	243 (34%)	136 (25%)	53 (28%)	324 (30%)	168 (30%)	133 (31%)	18 (33%)
2+	321 (45%)	141 (26%)	70 (37%)	389 (36%)	217 (39%)	148 (35%)	21 (39%)
Hypertension	243 (34%)	168 (31%)	52 (28%)	359 (33%)	193 (35%)	147 (35%)	14 (26%)
Diabetes	165 (23%)	136 (25%)	48 (25%)	251 (23%)	117 (21%)	115 (27%)	17 (31%)
Chronic pulmonary disease	95 (13%)	46 (8%)	19 (10%)	120 (11%)	86 (15%)	29 (7%)	4 (7%)
Grade 3/4 chronic kidney disease	76 (11%)	49 (9%)	24 (13%)	101 (9%)	58 (10%)	46 (11%)	1 (2%)
Renal failure	109 (15%)	70 (13%)	28 (15%)	151 (14%)	74 (13%)	75 (18%)	3 (6%)
Liver disease	35 (5%)	24 (4%)	5 (3%)	54 (5%)	28 (5%)	15 (4%)	0 (0%)
Peripheral vascular disorders	52 (7%)	25 (5%)	10 (5%)	67 (6%)	41 (7%)	25 (6%)	2 (4%)
Coagulopathy	30 (4%)	18 (3%)	4 (2%)	44 (4%)	29 (5%)	11 (3%)	1 (2%)
Pulmonary circulation disorders	28 (4%)	20 (4%)	8 (4%)	40 (4%)	24 (4%)	16 (4%)	0 (0%)
Obesity	52 (7%)	46 (8%)	11 (6%)	85 (8%)	49 (9%)	34 (8%)	3 (6%)
HIV/AIDS	2 (0%)	3 (1%)	1 (1%)	4 (0%)	2 (0%)	2 (0%)	1 (2%)
Rheumatoid arthritis/collagen vascular diseases	33 (5%)	20 (4%)	5 (3%)	48 (4%)	27 (5%)	14 (3%)	1 (2%)
Surgery in the year prior to 2/1/2020, N (%)	326 (45%)	135 (25%)	67 (35%)	393 (37%)	238 (43%)	141 (33%)	19 (35%)
Cancer‐related characteristics							
Active cancers, N (%)	719 (100%)	0 (0%)	109 (58%)	605 (56%)	324 (58%)	238 (56%)	43 (80%)
History of cancer, N (%)	0 (0%)	548 (100%)	80 (42%)	467 (44%)	235 (42%)	187 (44%)	11 (20%)
Cancer treatment in the 30 days prior to 2/1/2020, N (%)							
Immune checkpoint inhibitors	12 (2%)	0 (0%)	2 (1%)	10 (1%)	7 (1%)	4 (1%)	0 (0%)
Chemotherapy	39 (5%)	0 (0%)	7 (4%)	32 (3%)	20 (4%)	15 (4%)	2 (4%)
Targeted therapies	63 (9%)	0 (0%)	8 (4%)	55 (5%)	24 (4%)	31 (7%)	2 (4%)
Clinical endpoints							
Mortality, N (%)	109 (15%)	64 (12%)	35 (19%)	138 (13%)	93 (17%)	53 (12%)	10 (19%)
Hospital admissions, N (%)	583 (81%)	330 (60%)	146 (77%)	762 (71%)	419 (75%)	320 (75%)	45 (83%)
Invasive respiratory support, N (%)^a^	90 (19%)	48 (11%)	39 (24%)	98 (13%)	43 (12%)	71 (21%)	5 (26%)

^a^ Among those for whom data were available.

**FIGURE 1:**
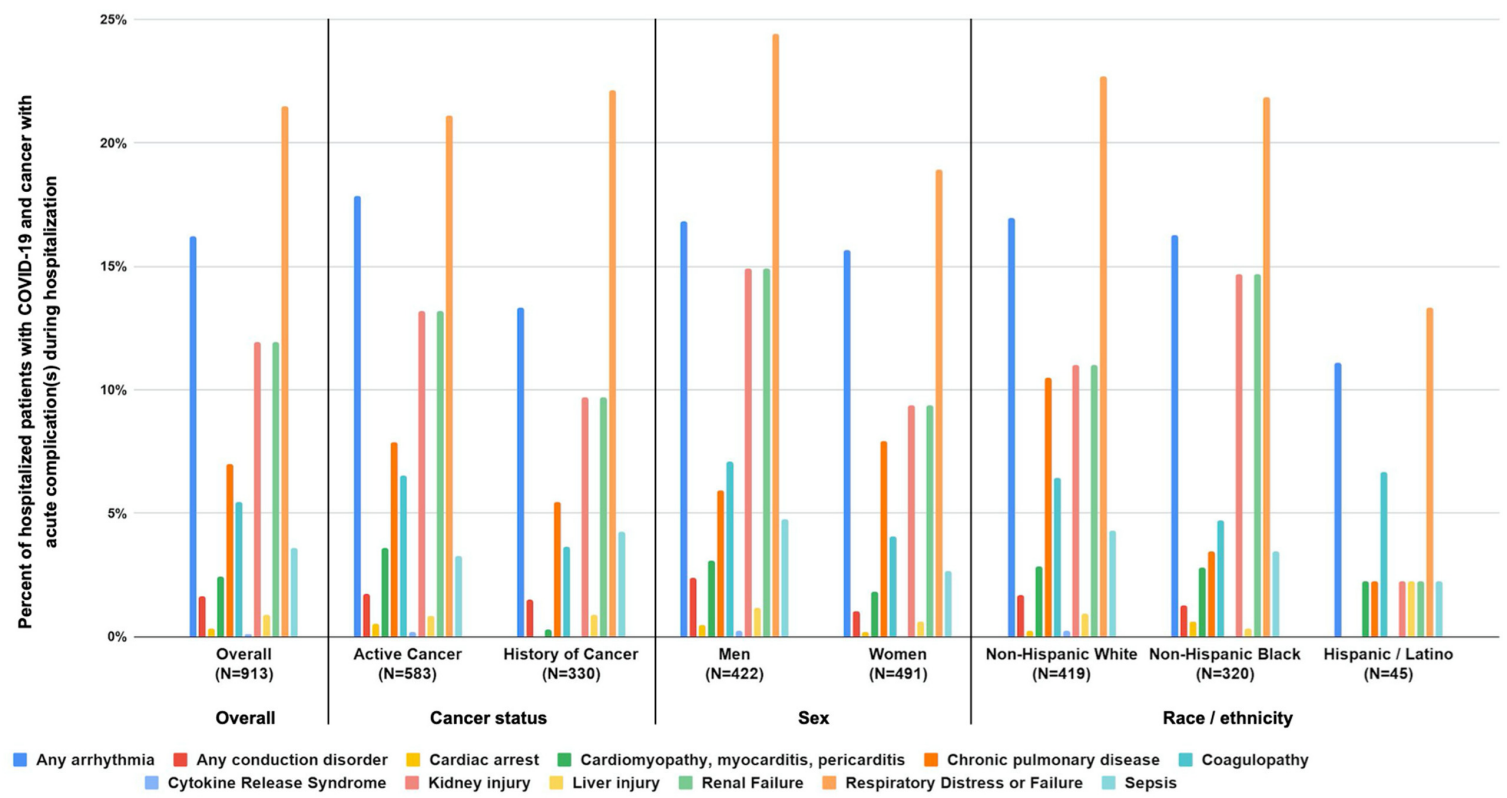
Distribution of acute complications during hospitalizations among patients with COVID‐19 and cancer who were hospitalized. The following acute complications are visualized in Figure 1: Any arrhythmia; Any conduction disorder; Cardiac arrest; Cardiomyopathy, myocarditis, pericarditis; Chronic pulmonary disease; Coagulopathy; Cytokine release syndrome; Kidney injury; Liver injury; Renal failure; Respiratory distress or failure; Sepsis

Admission diagnoses, treatments, and clinical outcomes for patients with COVID‐19 who were hospitalized are presented in Table S2. Within this cohort, the distributions of the 20 most common hospital admission diagnoses were similar for patients with active cancer and history of cancer. Breathing abnormalities were more common for males than females. Patients in zip codes with median household income below $30 000 were twice as likely as patients in zip codes with median household income above $30 000 to be hospitalized for pneumonia, fluid balance disorders, cough, respiratory failure, or acute renal failure. Additionally, NHB patients were more likely than other groups to be admitted for breathing abnormalities, pneumonia, fluid balance disorders, cough, acute renal failure or chronic kidney disease, and fever. NHB patients were more likely than other groups to be treated with hydroxychloroquine alone or in combination with azithromycin. In terms of acute COVID‐19 complications, patients in zip codes with median household income below $30 000 and males were more likely to experience respiratory distress or failure, as well as kidney and liver injury (Figure 1). Patients with a history of cancer and NHB patients were more likely to be released to self‐care, while patients in zip codes with median household income below $30 000 were more likely to expire while hospitalized (Table S3).

In adjusted multivariable analyses, we found that male sex, increasing age, living in a zip code with median household income below $30 000, history of pulmonary circulatory disorder, and recent treatment with immune checkpoint inhibitors or chemotherapy were all significantly associated with greater odds of all‐cause mortality (Table 3). While NHB and Hispanic/Latino race/ethnicity categories were not significantly associated with mortality compared to NHW, we found that a category that combined Asian/Native Hawaiian/Pacific Islander, other or unknown race/ethnicities, was associated with a lower all‐cause mortality.

**TABLE 3:** Odds ratios (OR) and 95% confidence intervals for all‐cause mortality among patients with cancer and COVID‐19

Characteristic	N (events)	OR (95% CI)
Sex		
Female	728 (77)	1
Male	539 (96)	1.5 (1.1, 2.2)
Age categories		
<60	442 (16)	1
60‐69	325 (36)	2.7 (1.5, 5.1)
≥70	500 (121)	7.6 (4.3, 13.5)
Race/ethnicity		
Non‐Hispanic white	559 (93)	1
Non‐Hispanic black	425 (53)	0.7 (0.4, 1.0)
Hispanic/Latino	54 (10)	1.3 (0.6, 2.9)
Asian/Hawaiian/PI/other/unknown	229 (17)	0.4 (0.2, 0.7)
Annual household income		
>30 k	1078 (138)	1
0‐30 k	189 (35)	2.0 (1.2, 3.3)
Comorbidities		
Charlson comorbidity score		
0	426 (38)	1
1	379 (39)	0.8 (0.5, 1.4)
2+	462 (96)	*1.6 (1.0, 2.6)*
Surgery in the year prior to 2/1/2020, N (%)	461 (64)	0.8 (0.5, 1.1)
Hypertension	411 (64)	0.8 (0.5, 1.1)
Coagulopathy	48 (11)	1.4 (0.7, 3.0)
Pulmonary circulation disorders	48 (15)	2.6 (1.3, 5.3)
Obesity	98 (6)	0.4 (0.2, 1.0)
Malignancy type		
Solid tumor site or neoplasm NOS	1077 (141)	1
Hematological malignancy	190 (32)	1.1 (0.7, 1.8)
Cancer status		
History of cancer	548 (64)	1
Active	719 (109)	1.1 (0.7, 1.6)
Cancer treatment in the 30 days prior to 2/1/2020 (active cancers)		
No treatment	1175 (152)	1
Immune checkpoint inhibitors	12 (6)	5.2 (1.2, 22.3)
Chemotherapy	39 (12)	3.3 (1.3, 8.1)
Targeted therapies	63 (12)	0.8 (0.3, 1.7)

## 4 DISCUSSION

Cancer treatment increases the potential for adverse health outcomes, including susceptibility to infections. As the COVID‐19 pandemic reached the United States, rapid and dramatic changes in cancer care delivery were instituted to mitigate exposure to patients with cancer. An understanding of factors associated with greater susceptibility to COVID‐19 infection and its sequelae can better inform care decisions and health care system interaction. In a large cohort of patients with cancer, patients with COVID‐19 were more likely to be NHB or to reside in zip codes with median household income below $30 000 than their counterparts who did not have COVID‐19. Among patients with COVID‐19, NHB patients or patients in zip codes with median household income below $30 000 were at greater risk of hospitalization and invasive respiratory support.

We estimated 14% mortality in patients with cancer and COVID‐19, compared to the 3% case‐fatality rate for COVID‐19 patients in the United States overall.^1^ The high mortality rate reported is consistent with previous reports^10^, ^13^, ^14^, ^17^, ^24^, ^27^ and consistent with the hypothesis that patients with cancer are at higher risk compared to patients without cancer. Estimated mortality rates vary, however, with a recent pooled analysis reporting the mortality for patients with both cancer and COVID‐19 as 25.6%, and other estimates ranging broadly from approximately 10% to 30%.^28^ Several factors may explain the variation in estimated mortality across studies. Differences in disease ascertainment likely exist among different medical systems and geographic regions and over time with rapidly changing clinical guidance. Additionally, the setting of diagnosis and evaluation is directly pertinent, as inpatient EMR data systems would be more likely to include populations with increased disease severity, a significant predictor of poor outcomes.^23^, ^24^ Alternatively, more aggressive screening or contact tracing may identify asymptomatic or minimally symptomatic cases, thus reducing the apparent case fatality rate. Differences in therapeutic approach (eg, remdesivir, corticosteroids, convalescent plasma) and baseline patient characteristics (sex, cancer history, comorbidities, disease severity) may also explain some of the variation in estimates across studies. Our study did not directly compare outcomes for COVID‐19 patients with cancer to a cohort of COVID‐19 patients without cancer.

The causes of increased COVID‐19 mortality in cancer patients are not fully known. Cancer patients are likely to be older and have a higher burden of comorbidities and frailty than the general population. Immunosuppressive cancer therapy may impair patients' ability to mount an effective antiviral immune response or mitigate deleterious multisystem effects. Consistent with this hypothesis, COVID‐19‐infected patients who have active cancer or recent cancer treatment have been found to have an increased risk of mortality,^24^, ^29^, ^30^ and in our study, recent chemotherapy and immune checkpoint inhibitor therapy were both independently associated with mortality. While some studies have reported an association between immune checkpoint inhibitor therapy and COVID‐19 severity or mortality,^11^, ^12^ others have not found evidence of such an association.^31^, ^32^, ^33^ It is unclear whether the association reflects the effects of immune checkpoint inhibitor therapy or the cancer etiology or represents the impact of the advanced nature of the cancer among patients receiving checkpoint inhibitor therapy, primarily utilized in patients with advanced disease. A prior history of pulmonary circulation disorders (eg, pulmonary embolism, pulmonary hypertension, diseases of pulmonary vessels) was also independently associated with an increased risk of all‐cause mortality in our study. The development of pulmonary embolism as a complication of COVID‐19 infection is well‐described, but to our knowledge, it has not previously been reported that a prior pulmonary circulation disorder is a risk for COVID‐19 mortality.

Although our study characterized the impact of race and socioeconomic status in one of the largest US cohorts of patients with cancer and COVID‐19 reported to‐date, similar associations have been reported in noncancer populations.^2^, ^3^, ^4^, ^5^, ^6^, ^22^ Further research is needed to establish the effects of the COVID‐19 pandemic felt disproportionally in under‐represented minority populations. Systematic health inequities as well as differences in access to health care, population density, multigenerational households, and the ability to telecommute are plausible explanations for how the complex interconnection between race and socioeconomic status might impact the risk from COVID‐19. Understanding the contribution of potential social and biological determinants of COVID‐19 severity requires study beyond the scope of this retrospective analysis. While we found that race/ethnicity were not significantly associated with higher all‐cause mortality in multivariable models, our findings highlight that race and socioeconomic factors identify a cancer population that is vulnerable to greater infection rates and morbidity. Addressing and supporting these vulnerabilities will be critical to minimize mortality risk from COVID‐19 in cancer patients.

Our cohort was characterized on the basis of EMR data from two distinct health care systems that serve predominantly Midwestern populations. These findings may not be generalizable to other populations. In addition, patient care received outside of these two systems (either for cancer or COVID‐19) was not captured, leading to potential misclassification of relevant medical history. Furthermore, assessment of comorbidities with ICD‐10 codes may have led to incomplete capture. At the time our data were collected, testing for COVID‐19 was primarily performed in symptomatic cases. Thus, our findings may not be extrapolated in an asymptomatic or minimally symptomatic population. Sample size limited our ability to evaluate effect modification by potentially relevant factors. Distribution of healthcare delivery sites is an important determinant of access to healthcare services. While the distance from a patient's current residence to the nearest inpatient or outpatient care site was not readily available for this rapid analysis, our informal investigation of the geographical distribution of COVID‐19 cases in our study population showed highest concentration in urban areas, which are typically characterized by shorter distance to care sites than rural areas. Despite the aforementioned limitations, this study demonstrates the strength of leveraging electronic data in a large integrated health care system. To our knowledge, this report of 1267 patients is one of the largest real‐world cohorts of COVID‐19 patients with cancer reported to date. With the ability to rapidly retrieve cases and associated clinical data, our methodology was able to confirm the results of previous studies, as well as identify new findings.

In conclusion, the emergence of a global pandemic caused by a novel and deadly pathogen has led to rapid and drastic changes in cancer care for public health reasons. RWD can be rapidly leveraged to characterize important demographic data and outcomes that may inform strategies to address urgent healthcare challenges such as COVID‐19. Our findings suggest that patients with cancer, especially those with active disease and/or comorbidities, are more vulnerable to the effects of COVID‐19, and heightened risks were observed in Non‐Hispanic black (NHB) patients and those living in zip codes with median household income below $30 000. Accurate and timely information is needed to formulate evidence‐based changes in the management of patients with cancer during and beyond the COVID‐19 pandemic^34^ to meet the needs of patients and enhance evidence‐guided clinical decision‐making.

## AUTHOR CONTRIBUTIONS

**Clara Hwang:** Writing‐original draft; writing‐review and editing. **Monika Izano:** Formal analysis; methodology; writing‐original draft; writing‐review and editing. **Michael Thompson:** Conceptualization; writing‐review and editing. **Shirish Gadgeel:** Conceptualization; writing‐review and editing. **James Weese:** Conceptualization; writing‐review and editing. **Tom Mikklesen:** Conceptualization; writing‐review and editing. **Andrew Schrag:** Data curation; formal analysis; software. **Mahder Teka:** Data curation; formal analysis; software. **Sheetal Walters:** Project administration. **Frank Wolf:** Conceptualization; visualization; writing‐review and editing. **Jonathan Hirsch:** Conceptualization; methodology. **Donna Rivera:** Writing‐review and editing. **Paul Kluetz:** Writing‐review and editing. **Harpreet Singh:** Writing‐review and editing. **Thomas Brown:** Conceptualization; methodology; supervision; writing‐review and editing.

## CONFLICT OF INTEREST

The authors have no conflicts of interest to disclose.

## ETHICS STATEMENT

This study was performed through a research collaboration agreement (RCA) between the FDA and Syapse. The RCA work has been performed under an exemption from the Office of the Chief Scientist (OCS) Human Subject Protection (HSP) Executive Officer at FDA.

## Supporting information


**Table S1.** Characteristics of the patients with cancer and COVID‐19, by sex
**Table S2**. Inpatient diagnoses, treatments, and acute complications of patients with COVID‐19 and cancer who were hospitalized.
**Table S3**. Distribution of discharge reasons among patients with COVID‐19 and cancer who were hospitalized.

## Data Availability

The data that support the findings of this study are available from the corresponding author upon reasonable request.
