# Supplementary material for: Rapid real‐world data analysis of patients with cancer, with and without COVID‐19, across distinct health systems
Source: Cancer Rep (Hoboken). 2021 May 20;4(5):e1388. doi: 10.1002/cnr2.1388 (PMC8209944; doi:10.1002/cnr2.1388)
Supplement: Supplementary file 1 — Table S1. Characteristics of the patients with cancer and COVID‐19, by sex Table S2. Inpatient diagnoses, treatments, and acute complications of patients with COVID‐19 and cancer who were hospitalized. Table S3. Distribution of discharge reasons among patients with COVID‐19 and cancer who were hospitalized. [file CNR2-4-e1388-s001.docx]

**SUPPLEMENTAL TABLES**

**Supplemental Table 1: Characteristics of the patients with cancer and COVID-19, by sex**

|  | | | **Men** | **Women** |
| --- | --- | --- | --- | --- |
|  |  |  | (N=539) | (N=728) |
| COVID-19 diagnosis, N (%) | | |  |  |
|  | Positive test result only | | 353 (84%) | 411 (84%) |
|  | ICD Code only | | 256 (61%) | 298 (61%) |
|  | Positive test result & ICD Code | | 235 (56%) | 273 (56%) |
| Clinical Characteristics | | |  |  |
|  | Comorbidities in the previous year, N (%) | |  |  |
|  |  | Charlson Comorbidity Index |  |  |
|  |  | Median, IQR | 1.0 (0, 2.0) | 1.0 (0, 2.0) |
|  |  | 0 | 142 (26%) | 284 (39%) |
|  |  | 1 | 168 (31%) | 211 (29%) |
|  |  | 2+ | 229 (42%) | 233 (32%) |
|  |  | Hypertension | 197 (37%) | 214 (29%) |
|  |  | Diabetes | 143 (27%) | 158 (22%) |
|  |  | Chronic pulmonary disease | 48 (9%) | 93 (13%) |
|  |  | Grade 3/4 chronic kidney disease | 63 (12%) | 62 (9%) |
|  |  | Renal failure | 95 (18%) | 84 (12%) |
|  |  | Liver disease | 33 (6%) | 26 (4%) |
|  |  | Peripheral vascular disorders | 46 (9%) | 31 (4%) |
|  |  | Coagulopathy | 33 (6%) | 15 (2%) |
|  |  | Pulmonary circulation disorders | 20 (4%) | 28 (4%) |
|  |  | Obesity | 32 (6%) | 66 (9%) |
|  |  | HIV/AIDS | 5 (1%) | 0 (0%) |
|  |  | Rheumatoid arthritis/collagen vascular diseases | 11 (2%) | 42 (6%) |
|  | Surgery in the year prior to 2/1/2020, N (%) | | 207 (38%) | 254 (35%) |
|  | Cancer-related characteristics | |  |  |
|  |  | Active Cancers, N (%) | 345 (64%) | 374 (51%) |
|  |  | History of Cancer, N (%) | 194 (36%) | 354 (49%) |
|  |  | Cancer treatment in the 30 days prior to 2/1/2020, N (%) |  |  |
|  |  | Immune checkpoint inhibitors | 3 (1%) | 9 (1%) |
|  |  | Chemotherapy | 25 (3%) | 1358 (2%) |
|  |  | Targeted therapies | 29 (5%) | 34 (5%) |
| Clinical Endpoints | | |  |  |
|  | Mortality, N (%) | | 96 (18%) | 77 (11%) |
|  | Hospital Admissions, N (%) | | 422 (78%) | 491 (67%) |
|  | Invasive respiratory support, N (%)^†^ | | 81 (21%) | 57 (11%) |

^†^ Among those for whom data was available

**Supplemental Table 2. Inpatient diagnoses, treatments, and acute complications of patients with COVID-19 and cancer who were hospitalized.**

|  | |  | ***Cancer Status*** | | ***Sex*** | | ***Median Household Income*** | | ***Race/ethnicity*** | | |
| --- | --- | --- | --- | --- | --- | --- | --- | --- | --- | --- | --- |
|  |  | **Overall (N=913)** | **Active Cancer (N=583)** | **History of Cancer (N=330)** | **Men (N=422)** | **Women (N=491)** | **0-30K (N=146)** | **>30K (N=762)** | **Non-Hispanic White (N=419)** | **Non-Hispanic Black (N=320)** | **Hispanic / Latino (N=45)** |
| Common Inpatient Admission Diagnoses, N (%) | |  |  |  |  |  |  |  |  |  |  |
|  | Abnormalities of Breathing | 256 (28%) | 148 (25%) | 108 (33%) | 133 (32%) | 123 (25%) | 52 (36%) | 203 (27%) | 95 (23%) | 109 (34%) | 4 (9%) |
|  | Pneumonia | 233 (26%) | 128 (22%) | 105 (32%) | 115 (27%) | 118 (24%) | 62 (42%) | 169 (22%) | 77 (18%) | 107 (33%) | 6 (13%) |
|  | Disorders of Fluid, Electrolyte and Acid-Base Balance | 156 (17%) | 78 (13%) | 78 (24%) | 62 (15%) | 94 (19%) | 40 (27%) | 114 (15%) | 46 (11%) | 72 (22%) | 4 (9%) |
|  | Cough | 140 (15%) | 85 (15%) | 55 (17%) | 67 (16%) | 73 (15%) | 36 (25%) | 102 (13%) | 48 (11%) | 60 (19%) | 5 (11%) |
|  | Respiratory Failure | 136 (15%) | 80 (14%) | 56 (17%) | 68 (16%) | 68 (14%) | 34 (23%) | 100 (13%) | 53 (13%) | 59 (18%) | 4 (9%) |
|  | Symptoms and Signs Involving the Circ and Resp Sys | 135 (15%) | 73 (13%) | 62 (19%) | 66 (16%) | 69 (14%) | 40 (27%) | 94 (12%) | 47 (11%) | 60 (19%) | 3 (7%) |
|  | Acute Kidney Failure | 127 (14%) | 76 (13%) | 51 (15%) | 76 (18%) | 51 (10%) | 38 (26%) | 87 (11%) | 43 (10%) | 58 (18%) | 3 (7%) |
|  | Fever | 122 (13%) | 73 (13%) | 49 (15%) | 52 (12%) | 70 (14%) | 25 (17%) | 96 (13%) | 36 (9%) | 59 (18%) | 2 (4%) |
|  | Hypertension | 121 (13%) | 64 (11%) | 57 (17%) | 61 (14%) | 60 (12%) | 31 (21%) | 89 (12%) | 48 (11%) | 49 (15%) | 4 (9%) |
|  | Abnormal Findings of Blood Chemistry | 102 (11%) | 58 (10%) | 44 (13%) | 54 (13%) | 48 (10%) | 18 (12%) | 81 (11%) | 34 (8%) | 37 (12%) | 3 (7%) |
|  | Pain in Throat and Chest | 100 (11%) | 58 (10%) | 42 (13%) | 64 (15%) | 36 (7%) | 18 (12%) | 81 (11%) | 47 (11%) | 32 (10%) | 2 (4%) |
|  | Long Term (Current) Drug Therapy | 95 (10%) | 56 (10%) | 39 (12%) | 49 (12%) | 46 (9%) | 22 (15%) | 73 (10%) | 37 (9%) | 35 (11%) | 3 (7%) |
|  | Type 2 Diabetes Mellitus | 95 (10%) | 53 (9%) | 42 (13%) | 43 (10%) | 52 (11%) | 32 (22%) | 62 (8%) | 26 (6%) | 44 (14%) | 5 (11%) |
| ***Supplemental Table 2, continued*** | |  | ***Cancer Status*** | | ***Sex*** | | ***Median Household Income*** | | ***Race/ethnicity*** | | |
|  |  | **Overall (N=913)** | **Active Cancer (N=583)** | **History of Cancer (N=330)** | **Men (N=422)** | **Women (N=491)** | **0-30K (N=146)** | **>30K (N=762)** | **Non-Hispanic White (N=419)** | **Non-Hispanic Black (N=320)** | **Hispanic / Latino (N=45)** |
| Common Inpatient Admission Diagnoses, N (%) | |  |  |  |  |  |  |  |  |  |  |
|  | Personal History of Other Diseases and Conditions | 93 (10%) | 55 (9%) | 38 (12%) | 54 (13%) | 39 (8%) | 29 (20%) | 64 (8%) | 37 (9%) | 37 (12%) | 0 (0%) |
|  | Chronic Kidney Disease (CKD) | 80 (9%) | 38 (7%) | 42 (13%) | 51 (12%) | 29 (6%) | 27 (18%) | 53 (7%) | 17 (4%) | 48 (15%) | 1 (2%) |
|  | Sepsis | 74 (8%) | 46 (8%) | 28 (8%) | 41 (10%) | 33 (7%) | 19 (13%) | 54 (7%) | 28 (7%) | 31 (10%) | 1 (2%) |
|  | Malaise and Fatigue | 73 (8%) | 47 (8%) | 26 (8%) | 46 (11%) | 27 (5%) | 13 (9%) | 59 (8%) | 35 (8%) | 22 (7%) | 3 (7%) |
|  | Abdominal and Pelvic Pain | 65 (7%) | 44 (8%) | 21 (6%) | 35 (8%) | 30 (6%) | 19 (13%) | 46 (6%) | 30 (7%) | 24 (8%) | 1 (2%) |
|  | Disorders of Mineral Metabolism | 64 (7%) | 41 (7%) | 23 (7%) | 33 (8%) | 31 (6%) | 25 (17%) | 39 (5%) | 27 (6%) | 26 (8%) | 1 (2%) |
|  | Heart Failure | 59 (6%) | 34 (6%) | 25 (8%) | 21 (5%) | 38 (8%) | 22 (15%) | 36 (5%) | 18 (4%) | 31 (10%) | 1 (2%) |
| Treatments, N (%) | |  |  |  |  |  |  |  |  |  |  |
|  | Vassopressors | 275 (30%) | 201 (34%) | 74 (22%) | 149 (35%) | 126 (26%) | 53 (36%) | 222 (29%) | 130 (31%) | 105 (33%) | 13 (29%) |
|  | Azithromycin alone | 124 (14%) | 78 (13%) | 46 (14%) | 55 (13%) | 69 (14%) | 14 (10%) | 110 (14%) | 73 (17%) | 32 (10%) | 9 (20%) |
|  | Hydroxychloroquine alone | 136 (15%) | 80 (14%) | 56 (17%) | 73 (17%) | 63 (13%) | 37 (25%) | 98 (13%) | 37 (9%) | 73 (23%) | 5 (11%) |
|  | Azithromycin & Hydroxychloroquine | 147 (16%) | 85 (15%) | 62 (19%) | 75 (18%) | 72 (15%) | 33 (23%) | 114 (15%) | 63 (15%) | 70 (22%) | 3 (7%) |
|  | Other antibiotic | 667 (73%) | 445 (76%) | 222 (67%) | 324 (77%) | 343 (70%) | 117 (80%) | 550 (72%) | 324 (77%) | 240 (75%) | 34 (76%) |
|  | Other antiviral | 82 (9%) | 63 (11%) | 19 (6%) | 45 (11%) | 37 (8%) | 16 (11%) | 66 (9%) | 38 (9%) | 31 (10%) | 7 (16%) |
|  | Famotidine | 214 (23%) | 150 (26%) | 64 (19%) | 91 (22%) | 123 (25%) | 45 (31%) | 169 (22%) | 97 (23%) | 87 (27%) | 12 (27%) |
|  | Tocilizumab | 38 (4%) | 24 (4%) | 14 (4%) | 26 (6%) | 12 (2%) | 5 (3%) | 33 (4%) | 17 (4%) | 14 (4%) | 2 (4%) |
|  | Remdesivir | 60 (7%) | 37 (6%) | 23 (7%) | 29 (7%) | 31 (6%) | 9 (6%) | 51 (7%) | 37 (9%) | 5 (2%) | 9 (20%) |
| Clinical Endpoints | |  |  |  |  |  |  |  |  |  |  |
|  | Mortality, N (%) | 169 (19%) | 107 (18%) | 62 (19%) | 95 (23%) | 74 (15%) | 35 (24%) | 134 (18%) | 90 (21%) | 53 (17%) | 10 (22%) |
| ***Supplemental Table 2, continued*** | |  | ***Cancer Status*** | | ***Sex*** | | ***Median Household Income*** | | ***Race/ethnicity*** | | |
|  |  | **Overall (N=913)** | **Active Cancer (N=583)** | **History of Cancer (N=330)** | **Men (N=422)** | **Women (N=491)** | **0-30K (N=146)** | **>30K (N=762)** | **Non-Hispanic White (N=419)** | **Non-Hispanic Black (N=320)** | **Hispanic / Latino (N=45)** |
| Clinical Endpoints | |  |  |  |  |  |  |  |  |  |  |
|  | Invasive respiratory support, N (%)^†^ | 81 (14%) | 90 (25%) | 48 (20%) | 81 (29%) | 57 (18%) | 39 (32%) | 98 (21%) | 43 (19%) | 71 (29%) | 5 (36%) |

^†^ Among those for whom data was available

**Supplemental Table 3. Distribution of discharge reasons among patients with COVID-19 and cancer who were hospitalized.**

|  | |  | ***Cancer Status*** | | ***Sex*** | | ***Median Household Income*** | | ***Race/ethnicity*** | | |
| --- | --- | --- | --- | --- | --- | --- | --- | --- | --- | --- | --- |
|  |  | **Overall (N=913)** | **Active Cancer (N=583)** | **History of Cancer (N=330)** | **Men (N=422)** | **Women (N=491)** | **0-30K (N=146)** | **>30K (N=762)** | **Non-Hispanic White (N=419)** | **Non-Hispanic Black (N=320)** | **Hispanic / Latino (N=45)** |
| Inpatient discharge reasons, N (%) | |  |  |  |  |  |  |  |  |  |  |
|  | Home/Self care | 246 (27%) | 145 (25%) | 101 (31%) | 102 (24%) | 144 (29%) | 41 (28%) | 203 (27%) | 89 (21%) | 107 (33%) | 4 (9%) |
|  | Expired | 52 (6%) | 30 (5%) | 22 (7%) | 32 (8%) | 20 (4%) | 17 (12%) | 35 (5%) | 21 (5%) | 24 (8%) | 3 (7%) |
|  | Home health | 51 (6%) | 28 (5%) | 23 (7%) | 23 (5%) | 28 (6%) | 9 (6%) | 42 (6%) | 18 (4%) | 17 (5%) | 2 (4%) |
|  | Skilled nursing facility | 32 (4%) | 13 (2%) | 19 (6%) | 13 (3%) | 19 (4%) | 6 (4%) | 25 (3%) | 14 (3%) | 8 (2%) | 1 (2%) |
|  | Hospice | 14 (2%) | 8 (1%) | 6 (2%) | 5 (1%) | 9 (2%) | 4 (3%) | 10 (1%) | 6 (1%) | 6 (2%) | 0 (0%) |
|  | Other acute inpatient hospital | 14 (2%) | 8 (1%) | 6 (2%) | 8 (2%) | 6 (1%) | 3 (2%) | 11 (1%) | 2 (0%) | 8 (2%) | 0 (0%) |
|  | Unknown | 14 (2%) | 8 (1%) | 6 (2%) | 12 (3%) | 2 (0%) | 3 (2%) | 11 (1%) | 5 (1%) | 6 (2%) | 1 (2%) |
|  | Against medical advise | 5 (1%) | 2 (0%) | 3 (1%) | 2 (0%) | 3 (1%) | 2 (1%) | 3 (0%) | 1 (0%) | 3 (1%) | 0 (0%) |
|  | Assisted living facility | 4 (0%) | 3 (1%) | 1 (0%) | 2 (0%) | 2 (0%) | 3 (2%) | 1 (0%) | 1 (0%) | 2 (1%) | 0 (0%) |
|  | Rehab facility | 1 (0%) | 1 (0%) | 0 (0%) | 0 (0%) | 1 (0%) | 0 (0%) | 1 (0%) | 0 (0%) | 0 (0%) | 0 (0%) |
|  | Missing | 480 (53%) | 337 (58%) | 143 (43%) | 223 (53%) | 257 (52%) | 58 (40%) | 420 (55%) | 262 (63%) | 139 (43%) | 34 (76%) |
